# Immediate effects of high-intensity laser therapy for nonspecific neck pain: a double-blind randomized controlled trial

**DOI:** 10.3389/fmed.2025.1550047

**Published:** 2025-04-11

**Authors:** Yuhua Xie, Yingxiu Diao, Dongyu Wu, Manxia Liao, Linrong Liao

**Affiliations:** ^1^Gannan Healthcare Vocational College, Ganzhou, China; ^2^Rehabilitation Medicine Center, The First Dongguan Affiliated Hospital, Guangdong Medical University, Dongguan, China; ^3^Department of Rehabilitation, Yixing JORU Rehabilitation Hospital, Wuxi, China; ^4^Department of Rehabilitation Medicine, The Second School of Clinical Medicine, Guangdong Medical University, Dongguan, China; ^5^Dongguan Key Laboratory of Intelligent Rehabilitation, Dongguan, China

**Keywords:** high-intensity laser therapy, nonspecific neck pain, muscle stiffness, randomized controlled trial, rehabilitation

## Abstract

**Objectives:**

The objective of this research was to assess the immediate effects of high-intensity laser therapy (HILT) on nonspecific neck pain (NNP) by evaluating outcome measures such as pain intensity, cervical active range of motion (ROM), stiffness of neck muscles, and functional disability.

**Methods:**

This clinical trial, which was conducted in a double-blind and randomized manner, involved patients diagnosed with NNP who were allocated either to either a HILT group (HILT + exercise) or a placebo group (placebo-laser therapy + exercise). The primary outcome measures encompassed pain intensity via the visual analogue scale (VAS), cervical active ROM, stiffness of neck muscles (splenius capitis, semispinalis capitis, and neck fascia), as well as functional disability via the neck disability index (NDI). Statistical evaluations were carried out using SPSS version 25.0, with a significance threshold established at *p* < 0.05.

**Results:**

A total of 28 individuals diagnosed with NNP were randomly allocated to either the HILT group (*n* = 14) or the placebo group (*n* = 14). Upon initial comparison, no significant differences (*p* > 0.05) were observed between the two groups. After treatment, both groups showed notable improvements in all outcome measures compared to baseline (*p* < 0.05); moreover, the HILT group demonstrated greater efficacy compared to the placebo group in terms of VAS scores (29.64 ± 8.43 mm, *p* = 0.001), cervical lateral flexion (right 22.46 ± 3.62°, *p* = 0.011; left 22.34 ± 2.74°, *p* = 0.034) and neck muscle shear modulus (splenius capitis muscle 22.48 ± 4.03 kPa, *p* = 0.001; semispinalis capitis muscle 23.50 ± 5.59 kPa, *p* = 0.028); however, no statistically significant differences (*p* > 0.05) were identified between the groups in cervical flexion, extension, rotation, neck fascia stiffness and NDI scores.

**Conclusion:**

HILT has immediate efficacy for NNP and may be considered as one of the alternative interventions for NNP.

**Clinical trial registration:**

http://www.chictr.org.cn/, identifier ChiCTR2200061008.

## 1 Introduction

Nonspecific neck pain (NNP) is defined as neck pain for which neither exact histopathological changes nor a clear aetiology can be identified (1). The point prevalence of NNP in the general adult population ranged from 21 to 50% (2, 3), and more than 50% of the patients with NNP were prone to relapse after 6 months or 1 year (4). Patients with NNP frequently experienced unilateral or bilateral soft tissue pain, stiffness, sensory-motor system dysfunction, and psychosocial burden (such as kinesiophobia, anxiety, depression) (5–8), which imposed an immeasurable socioeconomic burden worldwide (9). Therefore, it is crucial to provide effective clinical management for NNP.

Current clinical treatments for NNP remain limited. The main methods of treatment for NNP included medication as well as physical therapies such as exercise and physical agents, with wide variability in their clinical efficacy (1). Medication provided short-term pain relief, but long-term use had side effects such as increasing gastrointestinal bleeding and cardiovascular risk (10, 11). Although exercise was highly recommended in the clinical routine management of NNP (12, 13), there was insufficient patient adherence to repetitive therapy. Previous studies (14–16) suggested that the addition of physical agents such as laser therapy to exercise for musculoskeletal disorders (including neck pain) could have more improvements in pain and disability.

High-intensity laser therapy (HILT) was a non-invasive and safe laser therapy, and its analgesic effect stemmed from its dual modulation of deep tissues: the photochemical effect promoted adenosine triphosphate production (ATP) and inhibited inflammation through activation of the mitochondrial cytochrome c oxidase (17, 18); and the photothermal effect improved local blood circulation and reduced substance P release and central sensitization (19). Recent studies (20, 21) had further demonstrated that 1–3 sessions of HILT could provide immediate pain relief possibly through upregulating serum beta-endorphin levels. In addition, the study by Liechti et al. (22) showed that immediate analgesia could help patients cope with pain-related physical and psychological burden, and improved quality of life. The above evidence suggested that HILT had great potential for immediate analgesia in the clinical management of NNP.

To our knowledge, the number of studies of HILT for NNP was still limited. A meta-analysis encompassing 12 studies revealed that in comparison to a placebo, HILT was significantly effective in reducing pain intensity (SMD 2.12, 95% CI 1.24 to 3.00) and improving functional disability (SMD 1.73, 95% CI −0.05 to 3.54) in patients experiencing neck pain (23). Furthermore, only two studies (24, 25) indicated that the combination of HILT and exercise could alleviate pain and boost functionality in individuals with NNP, but these studies had several limitations, which included heterogeneity in HILT protocols (such as energy density, duration, and frequency), and only assessed efficacy in the short-term (2 weeks or even longer) after treatments. Kenareh et al. (25) found that 10 sessions of HILT immediately reduced pain and improved pain-related disability in patients with chronic NNP. Moreover, the study by Tuan et al. (26) noted that an immediate improvement in neck sprain and neck pain symptoms after a single HILT. However, there have been no immediate efficacy (within one day) trials of NNP after a single HILT treatment.

In summary, existing research has yet to demonstrate the immediate effectiveness of HILT in patients with NNP, thus necessitating additional investigation. Consequently, the primary aim of this trial was to examine the immediate effects of HILT on NNP, with the hypothesis that HILT would be superior compared to placebo in immediately enhancing pain relief, increasing cervical active range of motion (ROM) reducing muscle stiffness, and decreasing functional disability in individuals suffering from NNP.

## 2 Materials and methods

### 2.1 Study design and registration

The research was a randomized parallel-group trial that was conducted at the Rehabilitation Medicine Center of the First Dongguan Affiliated Hospital, Guangdong Medical University. It was a single-center investigation, which utilized a double-blinded and placebo-controlled design. The hospital’s Ethics Committee reviewed and granted approval for this protocol (Approval No. YS202204003), it was subsequently registered with the China Clinical Trial Registry^1^ under registration identifier ChiCTR2200061008. The reporting adhered to the Consolidated Standards of Reporting Trials (CONSORT) guidelines and recommendations for randomized trials (27). Prior to participating, all subjects received comprehensive information regarding the experimental procedures, and they provided written informed consent before being included in the study.

### 2.2 Subjects and sample sizes

Individuals expressing a willingness to take part in the research were evaluated to determine their suitability for inclusion and were given comprehensive details regarding the study. The inclusion criteria for this study were developed with the reference (1): (1) presence of significant pain, stiffness, or other discomfort in the neck region without numbness or radiating pain to the upper limbs; (2) neck pain in the acute phase (less than 30 days); (3) negative results for the foraminal compression test and the neck extension test; (4) pain score of 3 or higher on the visual analog scale (VAS); (5) age 18 years or older, with no restriction on gender; (6) no treatment received within one month of the current episode; (7) ability to read, write, and understand text, with good communication skills; (8) individuals who expressed their ability to participate voluntarily and could independently sign an informed consent form. The criteria for exclusion included the following: (1) history of cervical spine trauma or surgery; (2) significant cervical disc herniation, spinal narrowing, cervical nerve root compression, or cervical spinal cord dysfunction; (3) history of severe and frequent migraines, fibromyalgia, shoulder diseases, rheumatic or rheumatoid diseases, severe osteoporosis, mental illnesses, significant spinal deformities, or serious cardiovascular, pulmonary, or neurological diseases; (4) presence of fever, infection, tumors, cancer, or severe systemic diseases; (5) cognitive or emotional disorders; (6) pregnant or breastfeeding women.

The sample size for the NNP patients to be recruited in this study was calculated based on the sample size formula for comparing the means of two independent samples (as shown in the formula below). Assuming a power of 1 − *β* = 0.8, a significance level of *α* = 0.05, with equal sample sizes in both groups, and using a two-tailed test. The sample size calculation for this study was determined using the VAS. According to previous study (20, 28), 𝛿 = 15, 𝜎 = 10.90, *Z*_*α*/2_ = 1.96, *Z_β_* = 0.84. After preliminary calculations, the total sample size was determined to be 18. However, considering the shedding rate 20–30% and the potential challenges during the trial, such as participant dropout or failure to meet study requirements, the sample size was increased to address these possibilities. Furthermore, to ensure sufficient statistical power to detect differences between groups, additional participants were included. Consequently, the final total sample size was determined to be 28.


$$n=\frac{2{\left(Z_{\alpha /2}+Z_{\beta }\right)}^{2}\times \sigma^{2}}{\delta^{2}}$$

𝛿: the mean difference, 𝜎^2^: the overall variance.

### 2.3 Randomization and blinding

Patients were randomly assigned to either the HILT group or the placebo group. Randomization was performed using SPSS statistical software, employing a 1:1 allocation ratio stratified by center and utilizing random block sizes of four. The randomization sequence was generated by an independent researcher who was not involved in participant recruitment or treatment. Numbered cards indicating group allocation were placed in opaque, sealed envelopes by the independent researcher. These envelopes were then opened by the intervention researchers, who administered the designated treatment program based on the assigned group. Each patient in two groups was blinded to their group assignment throughout the study. The therapists were aware of the grouping of the patients but not of the specific parameter settings, as laser parameters were preset by an independent researcher, and the therapists only performed standardized procedures. Assessment of all patients was done by another researcher who was not aware of the grouping.

### 2.4 Interventions

#### 2.4.1 High-intensity laser therapy

Both groups used the Lightforce*LT S-1500 laser therapy device produced by LiteCure, United States, for laser therapy. The laser type was Class IV, with a solid-state laser wavelength of 980/810 nm, operating in continuous mode, and the probe was a large spherical probe. The treatment provider set the corresponding laser treatment parameters for each patient according to their group assignment (24). Table 1 shows the specific treatment parameters for both groups’ laser therapies. The placebo in the control group was similar in operation to the HILT in the treatment group, with the main difference being that the laser output was turned off during the placebo.

**Table 1:** Parameters of laser therapy for two groups.

Groups	Treatment area	Scanning mode	Total energy dose (J)	Power (W)	Continuous scanning time	Irradiation time
HILT	① Paravertebral region of both cervical vertebrae, interscapular region, trapezius, sternocleidomastoid, and pain trigger points	Fast manual landscape and portrait scanning (100 cm^2^/30 s)	1,025	8	2 min	6 min/session
	② 6 pain trigger points (3 on each side: the midpoint of C_7_ and the acromion; the interscapular region/T_1_ paraspinal region; the C_2_ paraspinal region)	Small area scanning of pain trigger points	750 (125 J per pain trigger point)	8	15 s per pain trigger point	
	③ Paravertebral region of both cervical vertebrae, interscapular region, trapezius, sternocleidomastoid, and pain trigger points	Slow manual landscape and portrait scanning (100 cm^2^/60 s)	1,025	8	2 min	
Placebo	① Paravertebral region of both cervical vertebrae, interscapular region, trapezius, sternocleidomastoid, and pain trigger points	Fast manual landscape and portrait scanning (100 cm^2^/30 s)	0	0	2 min	6 min/session
	② 6 pain trigger points (3 on each side: the midpoint of C_7_ and the acromion; the interscapular region/T_1_ paraspinal region; the C_2_ paraspinal region)	Small area scanning of pain trigger points	0	0	15 s per pain trigger point	
	③ Paravertebral region of both cervical vertebrae, interscapular region, trapezius, sternocleidomastoid, and pain trigger points	Slow manual landscape and portrait scanning (100 cm^2^/60 s)	0	0	2 min	

HILT, high-intensity laser therapy.

During the treatment, the patient was seated in an upright position, with their lower back straight and against the chair back, arms naturally hanging by the sides of the body, and the neck and shoulder area fully exposed. The treatment provider used an alcohol swab to disinfect the treatment area and the large spherical probe, then placed the laser probe perpendicular to the skin, making light contact with the skin. Finally, the laser emission button was either activated or deactivated (activated for HILT, deactivated for placebo), and the laser treatment was administered in phases to the patient.

#### 2.4.2 Cranio-cervical exercises therapy

After laser therapy, all patients underwent cranio-cervical exercises therapy lasts approximately 30 min, with the following specific steps:

*Cranio-cervical muscle strength training* (29, 30): the patient begins in a quadruped position with the spine aligned in a straight line, arms positioned directly beneath the shoulders, and knees directly under the hips. The exercise starts with the patient slowly performing cranio-cervical flexion (range of motion less than 10°) while maintaining the cervical spine in a neutral position, followed by a gradual return to the neutral posture. The patient then performs slow cervical extension (range less than 20°), left rotation, and right rotation (each rotation limited to less than 40°). Each movement is repeated 10 times per set, with a 10-s rest between sets, for a total of three sets.

*Cranio-cervical extensor muscle training* (31, 32): the patient assumes a quadruped position with the spine aligned in a straight line, arms directly beneath the shoulders, and knees positioned under the hips. The therapist applies resistance at the spinous process of the fourth cervical vertebra to activate the deeper cervical extensor muscles (e.g., semispinalis capitis) while minimizing activation of superficial extensor muscles (e.g., splenius capitis). The patient maintains a cranio-cervical neutral position for 10 s, rests briefly, and repeats this process 10 times. Subsequently, resistance is applied at the spinous process of the second cervical vertebra, and the patient performs slow cranio-cervical extension 10 times per set, with a 10-s rest between repetitions. This sequence is repeated for a total of three sets.

*Deep cervical flexor training* (33, 34): this exercise uses a biofeedback pressure device (Stabilizer Pressure Biofeedback, Model JHY, United States). The patient lies in a supine position with knees bent and the head maintained in a neutral position to avoid backward tilting or chin protrusion. A towel may be placed under the occiput for support. The folded pressure bag, positioned at the neck near the occiput and secured with a button, is inflated to 20 mmHg. The patient is instructed to place the tongue on the roof of the mouth, keeping the teeth slightly apart to prevent compensatory contraction of the platysma or hyoid muscles. The patient then performs a gentle nodding action, aiming to maintain the pressure within the range of 22–24 mmHg. If the patient can maintain stability, they hold the position for 10 s, relax for 10 s, and repeat the sequence 10 times per set, completing three sets in total.

### 2.5 Outcomes

Data on sociodemographic factors such as age, gender, duration of complaints, height, and weight were collected from all participants in the study. The main outcome measure for this research was pain intensity, assessed using the visual analogue scale (VAS). Secondary outcomes included cervical active ROM, neck tissue stiffness evaluated through the shear wave elastography (SWE), and functional disability assessed with the neck disability index (NDI). All participants underwent assessments both before treatment and 2 h after treatment, conducted by researchers, based on the peak action window of HILT photo-biomodulation (17) and prior clinical protocols (21).

#### 2.5.1 Primary outcome

##### 2.5.1.1 Pain intensity

Pain intensity was evaluated utilizing the visual analogue scale (VAS), a measurement that varied from 0 to 100, with 0 representing “no pain” and 100 indicating “the worst possible pain” (35). Previous study has confirmed the VAS as a trustworthy and efficient instrument for quantifying acute pain, demonstrating high reliability (ICC = 0.96–0.98), and the minimum clinically important difference (MCID) of the VAS was defined as a decrease of greater than or equal to 10 mm, and changes below this value were considered meaningless (36).

#### 2.5.2 Secondary outcomes

##### 2.5.2.1 Cervical active range of motion

Cervical active ROM was measured with the patient seated upright, maintaining a straight back and resting against the chair. Arms were positioned naturally at their sides, feet placed shoulder-width apart and flat on the floor, and knees bent at a 90-degree angle. The patient was instructed to actively perform head and neck movements, including flexion, extension, lateral flexion (to the left and right), and rotation (to the left and right). The movement was stopped if the patient experienced pain or discomfort; otherwise, the patient moved to the maximum achievable ROM. A goniometer was used to measure ROM in each direction. Each motion was measured three times, and the average of the three values was recorded. Cervical active ROM measurements demonstrated excellent reliability (37).

##### 2.5.2.2 Neck tissues stiffness

The stiffness of neck tissues was assessed using shear wave elastography (SWE), which has been shown to have high test-retest reliability for measuring the elastic modulus of superficial cervical extensor muscles (38). Shear modulus values of the splenius capitis, semispinalis capitis, and nuchal fascia were measured for all patients using the Canon Aplio i800 ultrasound diagnostic device, equipped with a high-frequency linear array transducer (model i18LX5) operating at 5–18 MHz. The device used default musculoskeletal settings for SWE mode, with the shear modulus measurement range set to 0–100 kPa.

The patient was positioned upright, with their back straight and resting against the chair, arms relaxed at their sides, feet shoulder-width apart and flat on the floor, knees bent at 90 degrees, and the head and neck in a neutral position to allow full cervical region exposure. Routine gray-scale ultrasounds were first performed to locate the spinous process of the second cervical vertebra using a transverse view. The transducer was then rotated 90 degrees clockwise to a longitudinal position (39), approximately 1.5 cm lateral to the right of the spinous process, and adjusted to align parallel to the posterior median line of the spine for two-dimensional imaging of the cervical muscles.

**Figure 1:**
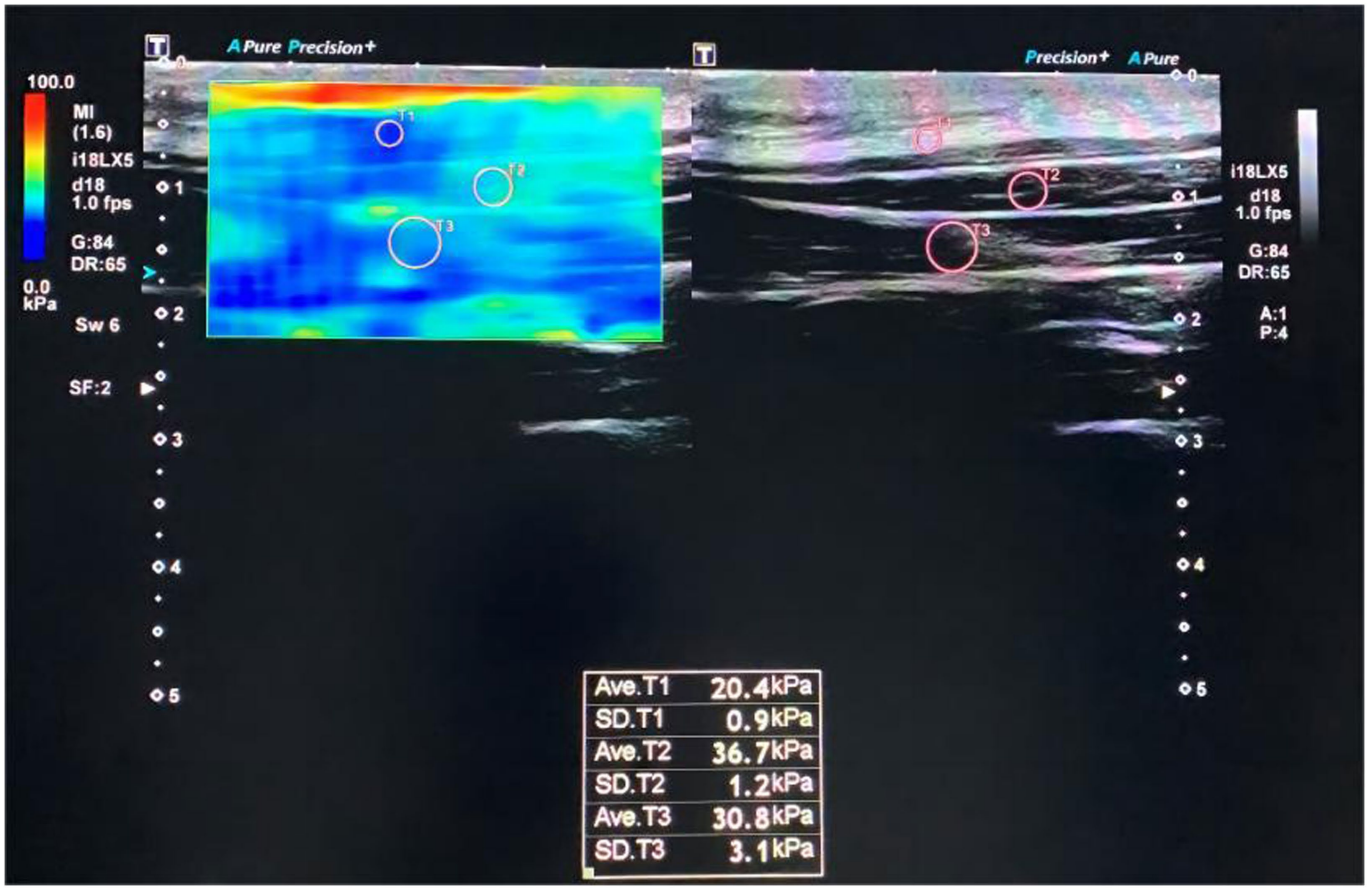
Shear wave elastography maps of neck tissues.

Once clear and stable images were obtained, the SWE mode was activated to capture elastography images. The left side of the split screen displayed elastography, while the right side displayed two-dimensional imaging. The region of interest (ROI) was adjusted to include the nuchal fascia, upper trapezius, splenius capitis, and semispinalis capitis. Once stable dual images were observed for 3–4 s, the frame was frozen. Circular sample frames of appropriate size were selected for the nuchal fascia, splenius capitis, and semispinalis capitis in each patient’s image. The device’s built-in software then calculated the shear modulus values, as shown in Figure 1. During measurements, patients were instructed to breathe naturally and maintain the initial posture. Each measurement was repeated three times, and the average value was recorded for statistical analysis.

##### 2.5.2.3 Functional disability

The evaluation of functional disability in individuals with NNP was conducted using the Chinese adaptation of the NDI. This assessment tool is comprised of 10 items that are categorized into two sections: symptoms related to neck pain and the ability to perform daily activities. Each item can receive a score ranging from 0 to 5, resulting in a maximum achievable score of 50. Elevated scores reflect an increased level of cervical functional disability. The Chinese version of the NDI has been validated as a reliable, effective, and sensitive tool for assessing cervical functional disability in patients with neck pain (40).

### 2.6 Statistical analysis

Statistical evaluations in this trial adhered to the intention-to-treat (ITT) protocol. All analyses were executed via SPSS 25.0 (IBM Corp, Armonk, NY). Demographic characteristics, including sex, were expressed as frequency counts and proportions (%) with intergroup differences analyzed using Pearson’s chi-square test. Normality assumptions for continuous variables were verified through the Shapiro–Wilk method. Variables conforming to normal distribution were reported as mean ± standard deviation (SD), with independent *t*-tests comparing baseline differences between groups and paired t-tests evaluating pre-post intervention changes. For skewed datasets, nonparametric approaches were adopted: central tendency and dispersion were summarized using median and interquartile ranges (IQR), while the Mann–Whitney *U* test and Wilcoxon signed-rank test were employed for cross-group and longitudinal comparisons, respectively. Statistical significance thresholds were set at *p* < 0.05.

## 3 Results

### 3.1 Patient flow and recruitment

**Figure 2:**
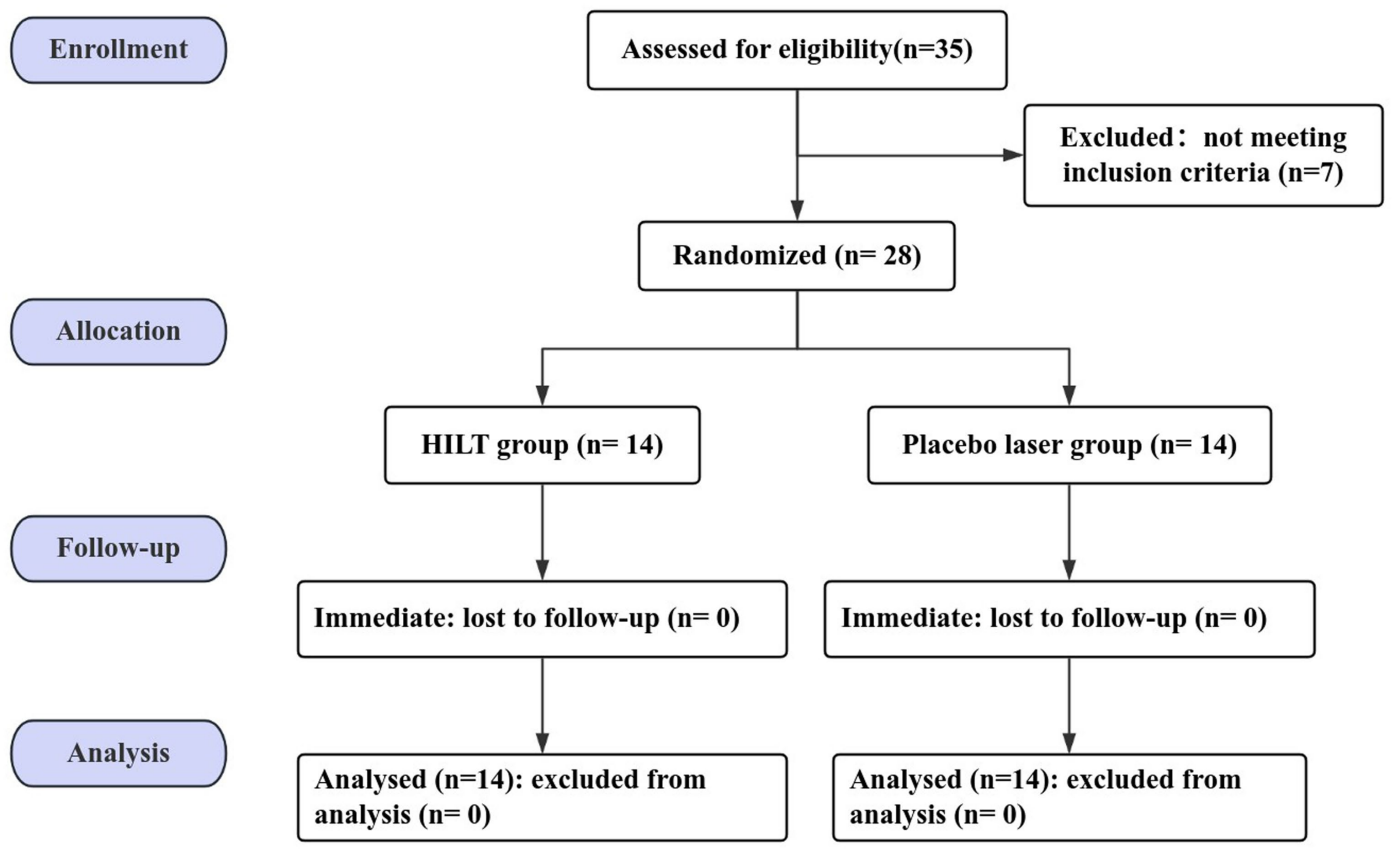
Participant flow diagram.

A total of 35 patients with neck pain were screened for enrolment eligibility and finally 28 patients with NNP who met the inclusion criteria and voluntarily agreed to participate in this study. The CONSORT flow diagram was shown in Figure 2.

### 3.2 Demographic characteristics

The 28 NNP patients included in the study were randomized to the HILT group and the placebo group. Comparison of the clinical characteristics between two groups revealed that there were no statistically significant differences (*p* > 0.05), as shown in Table 2.

**Table 2:** Clinical and demographic characteristics of the participants in two groups.

Characteristics	HILT group (*n* = 14)	Placebo group (*n* = 14)	*p*-value
Age (year), mean ± SD	26.43 ± 4.78	25.86 ± 4.28	0.742
Gender (female/male), *n*/*n*	8/6	7/7	0.705
Height (cm), mean ± SD	165.36 ± 9.18	167.43 ± 9.36	0.560
Weight (kg), mean ± SD	55.43 ± 9.53	60.71 ± 13.32	0.238
Pain duration (day), mean ± SD	3.71 ± 2.02	4.07 ± 1.86	0.630
VAS (mm), mean ± SD	53.57 ± 9.08	54.64 ± 8.43	0.749
Cervical AROM (°), mean ± SD			
Flexion	33.84 ± 9.06	32.05 ± 7.17	0.566
Extension	41.36 ± 11.08	38.71 ± 10.15	0.515
Right lateral flexion	19.17 ± 3.64	17.42 ± 3.96	0.233
Left lateral flexion	19.42 ± 3.35	18.19 ± 4.62	0.428
Right rotation	49.80 ± 7.78	51.30 ± 7.26	0.398
Left rotation	50.07 ± 5.76	52.46 ± 6.23	0.303
Neck tissues stiffness (kPa), mean ± SD			
Splenius capitis muscle	31.72 ± 7.86	31.85 ± 6.44	0.964
Semispinalis capitis muscle	31.30 ± 9.52	31.59 ± 5.62	0.920
Neck fascia	32.13 ± 9.32	28.36 ± 8.61	0.276
NDI (score), mean ± SD	11.64 ± 2.76	10.07 ± 2.95	0.157

*p-value was obtained by comparing the HILT group with the placebo group, and p < 0.05 indicated that the difference was statistically significant. HILT, high-intensity laser therapy; VAS, visual analogue scale; AROM, active range of motion; NDI, neck disability index.*

### 3.3 The result of primary outcome

**Figure 3:**
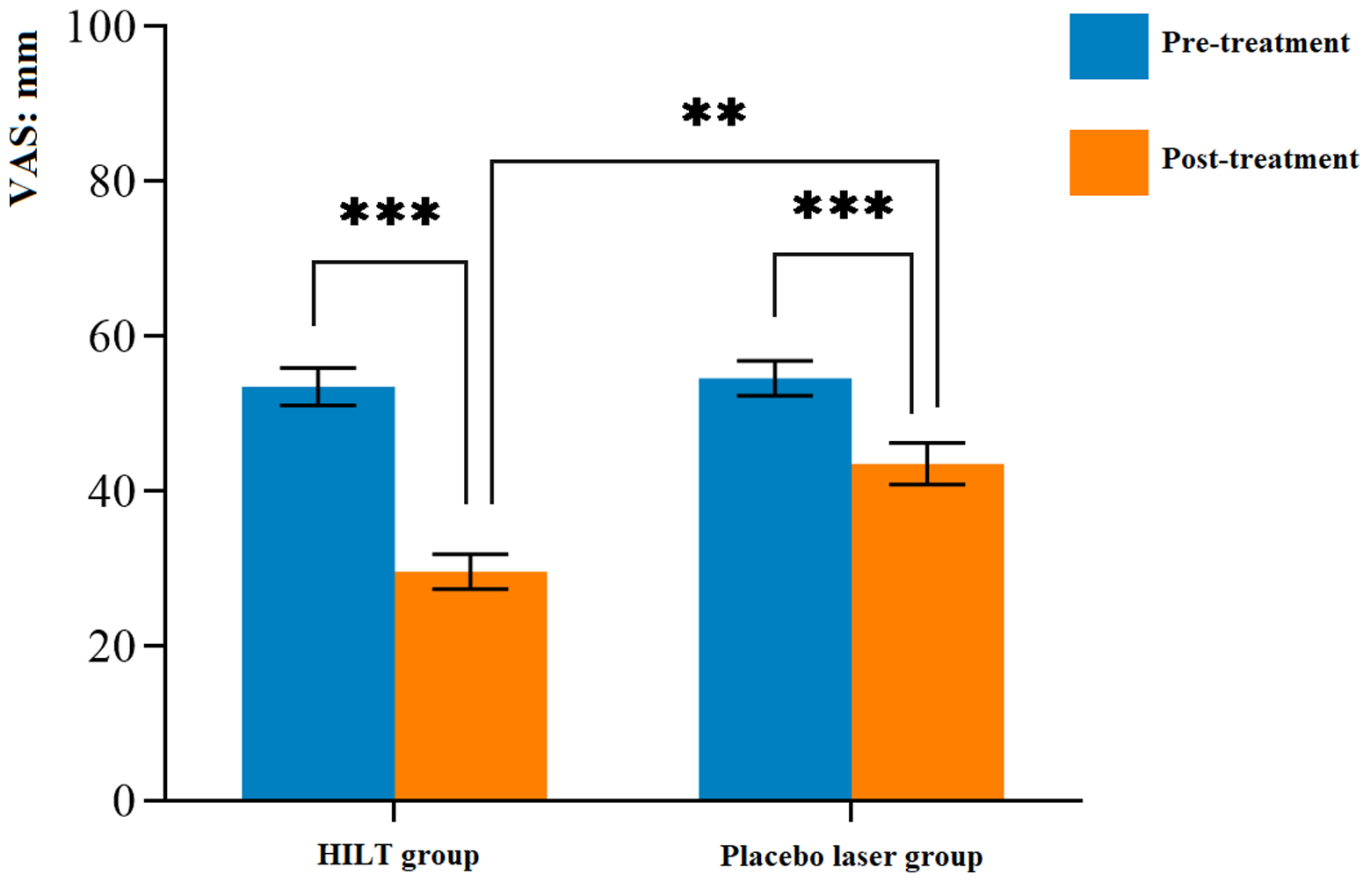
Mean and standard deviation of the pain intensity. **p < 0.01 and ***p < 0.001; VAS, visual analogue scale; HILT, high-intensity laser therapy.

The pain intensity of all patients were evaluated using the VAS, with detailed statistical data presented in Table 3 and Figure 3. After treatment, both groups exhibited a significant reduction in VAS scores compared to their pre-treatment levels (*p* < 0.05). Prior to treatment, no significant difference was observed between the groups (*p* > 0.05). After treatment, the HILT group demonstrated a notably larger decrease in VAS scores than the placebo group (29.64 ± 8.43 mm, *p* = 0.001). Notably, the mean reduction exceeded the MCID threshold of 10 mm. None of the patients had any adverse effects during the trial.

**Table 3:** The results of comparison of variables in two groups.

Variables	Pre-treatment (mean ± SD)		Post-treatment (mean ± SD)		*p*-value (within-group)		*p*-value (between-group)
	HILT group (*n* = 14)	Placebo group (*n* = 14)	HILT group (*n* = 14)	Placebo group (*n* = 14)	HILT group	Placebo group	
VAS (mm)	53.57 ± 9.08	54.64 ± 8.43	29.64 ± 8.43	43.57 ± 10.08	<0.001	<0.001	**0.001**
Cervical AROM (°)							
Flexion	33.84 ± 9.06	32.05 ± 7.17	38.24 ± 9.56	33.82 ± 7.03	<0.001	<0.001	0.175
Extension	41.36 ± 11.08	38.71 ± 10.15	46.15 ± 9.61	40.85 ± 10.66	<0.001	0.005	0.178
Right lateral flexion	19.17 ± 3.64	17.42 ± 3.96	22.46 ± 3.62	18.51 ± 4.00	<0.001	<0.001	**0.011**
Left lateral flexion	19.42 ± 3.35	18.19 ± 4.62	22.34 ± 2.74	19.31 ± 4.26	<0.001	0.001	**0.034**
Right rotation	49.80 ± 7.78	51.30 ± 7.26	52.46 ± 8.53	53.45 ± 5.98	0.001	<0.001	0.723
Left rotation	50.07 ± 5.76	52.46 ± 6.23	53.17 ± 5.25	53.83 ± 5.86	0.001	<0.001	0.757
Neck tissues stiffness (kPa)							
Splenius capitis muscle	31.72 ± 7.86	31.85 ± 6.44	22.48 ± 4.03	28.90 ± 5.19	<0.001	<0.001	**0.001**
Semispinalis capitis muscle	31.30 ± 9.52	31.59 ± 5.62	23.50 ± 5.59	28.29 ± 5.27	<0.001	<0.001	**0.028**
Neck fascia	32.13 ± 9.32	28.36 ± 8.61	22.90 ± 5.13	26.46 ± 7.54	<0.001	0.001	0.156
NDI (score)	11.64 ± 2.76	10.07 ± 2.95	8.36 ± 2.24	9.50 ± 2.95	<0.001	0.001	0.259

*p < 0.05 indicated a significant difference. Bold values in table indicate statistically significant differences between groups. VAS, visual analogue scale; AROM, active range of motion; NDI, neck disability index; HILT, high-intensity laser therapy.*

### 3.4 The results of secondary outcomes

**Figure 4:**
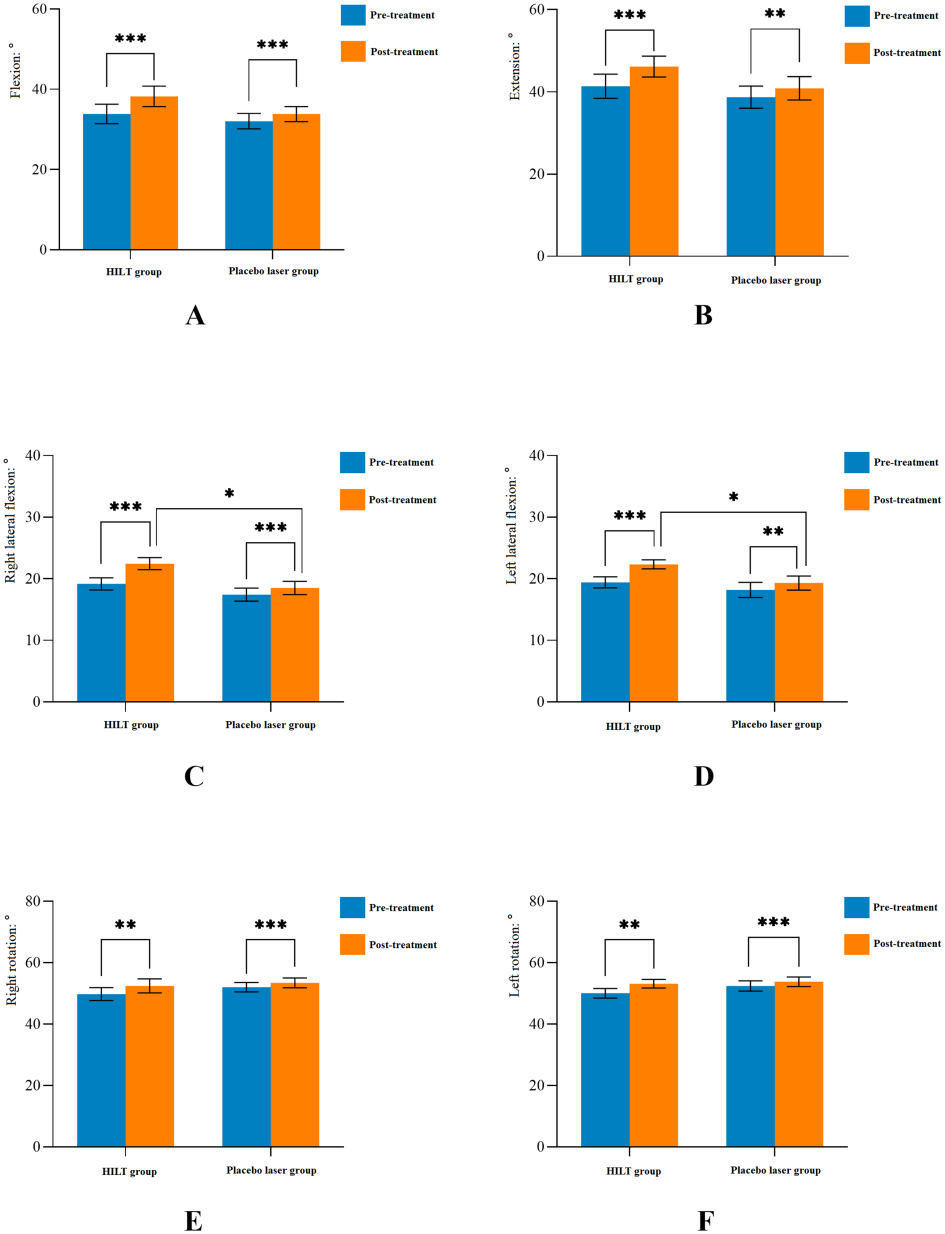
Mean and standard deviation of cervical active range of motion. ^*^p < 0.05, ^**^p < 0.01, and ^***^p < 0.001; HILT, high-intensity laser therapy.

**Figure 5:**
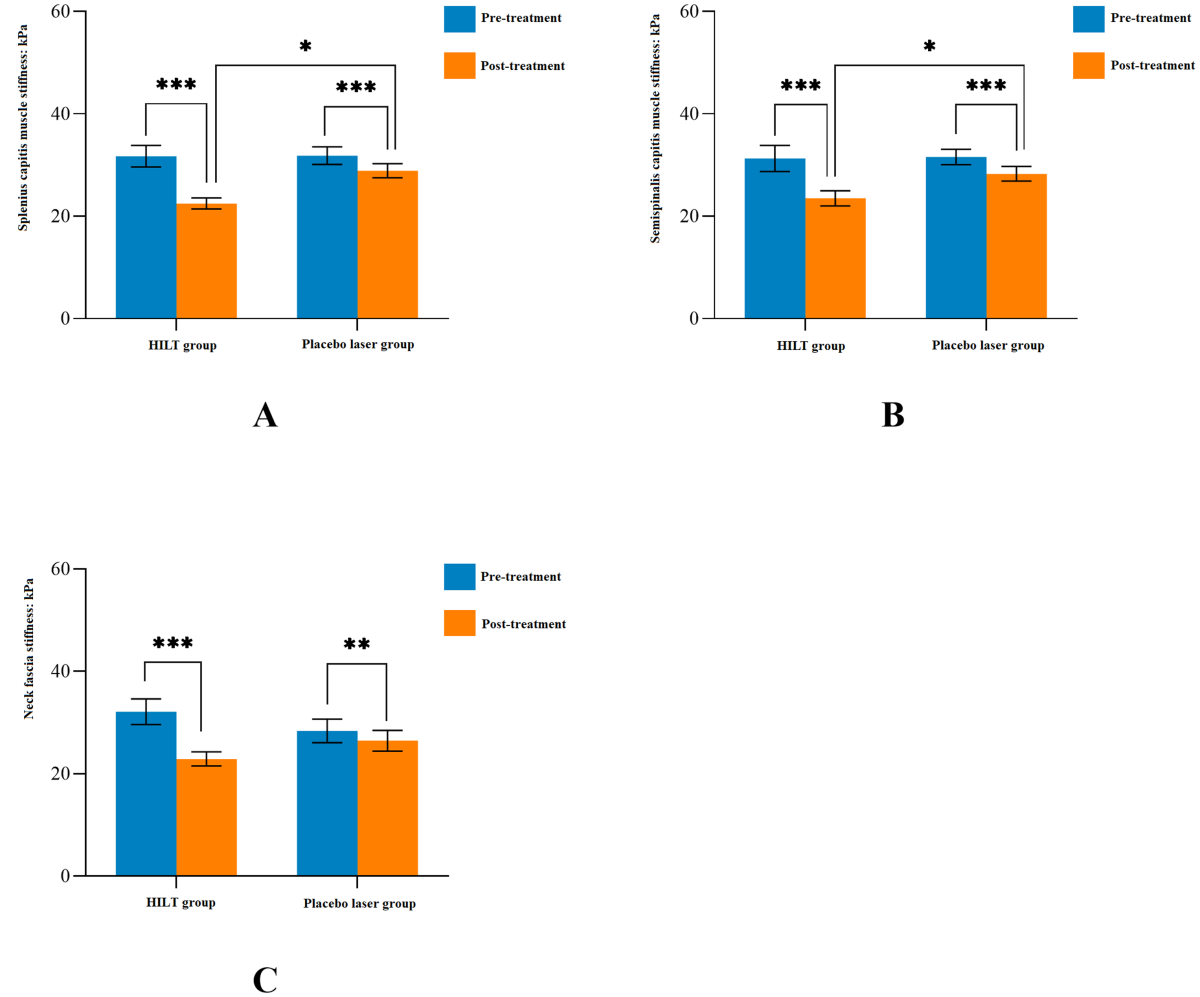
Mean and standard deviation of neck tissues stiffness. ^*^p < 0.05, ^**^p < 0.01, and ^***^p < 0.001; HILT, high-intensity laser therapy.

**Figure 6:**
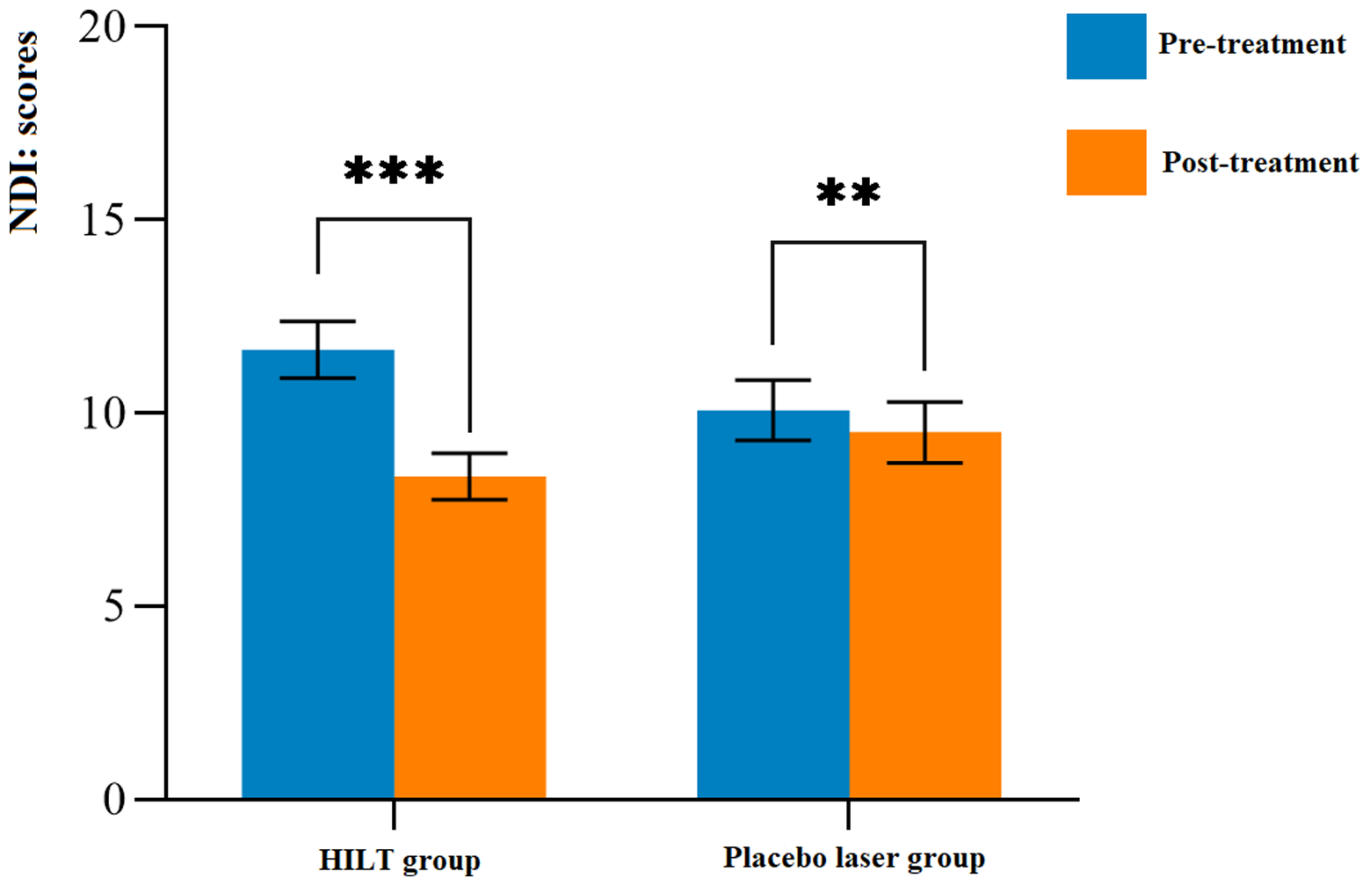
Mean and standard deviation of functional disability. **p < 0.01 and ***p < 0.001; NDI, neck disability index; HILT, high-intensity laser therapy.

The detailed statistical results of the secondary outcomes are shown in Table 3. Significant differences were observed in cervical ROM, neck tissue stiffness (splenius capitis muscle, semispinalis capitis muscle, and neck fascia), and NDI scores between pre-treatment and post-treatment in both groups (*p* < 0.05). Before treatment, there were no statistically significant differences between the two groups in terms of cervical ROM, neck tissue stiffness, or NDI scores (*p* > 0.05). After treatment, the HILT group demonstrated greater effectiveness in improving cervical lateral flexion (right: 22.46 ± 3.62°, *p* = 0.011; left: 22.34 ± 2.74°, *p* = 0.034, Figure 4) and reducing neck muscle stiffness (splenius capitis muscle: 22.48 ± 4.03 kPa, *p* = 0.001; semispinalis capitis muscle: 23.50 ± 5.59 kPa, *p* = 0.028, Figure 5) compared to the placebo group. However, no statistically significant differences were observed between the two groups in cervical flexion, extension, rotation, neck fascia stiffness, or NDI scores (*p* > 0.05, Figure 6).

## 4 Discussion

The main objective of this study was to investigate the immediate efficacy of HILT in patients with NNP. The results showed that the HILT combined with exercise had better immediate efficacy in improving pain, cervical lateral flexion ROM, and stiffness of superficial cervical muscles (splenius capitis muscle, semispinalis capitis muscle) in patients with NNP, but no significant differences were observed in other outcomes, which implied that there was a synergistic effect between HILT and exercise in the clinical treatment of patients with NNP, and that HILT could benefit the immediate efficacy of exercise.

HILT combined with exercise was more effective in reducing pain in patients with NNP, this finding that was consistent with the results of previous studies (24, 25). It could be attributed to the synergistic interaction between photobiomodulation of HILT and exercise-induced neurophysiological adaptations (14). HILT delivered targeted photochemical effect, which enhanced cellular mitochondrial ATP synthesis to promote tissue healing and regeneration, and suppressed pro-inflammatory cytokines and nociceptive transmission (17–19, 41). In addition, the photothermal effect of HILT could increase local microcirculation, and stimulated immunological processes and nerve regeneration, accelerating collagen synthesis and tissue repair (19, 42). Concurrently, the efficacy of exercise in this study may enhance proprioceptive feedback from neck mechanoreceptors, thereby normalising abnormal motor control patterns (43, 44), and promote exercise-induced release of β-endorphins and brain-derived neurotrophic factor, which may suppress central sensitization, as evidenced by reduced temporal summation of pain in NNP patients following motor control training (6).

The patients with neck pain may have an abnormal psychology of fear of movement, which leads to selective braking of the neck in order to avoid causing or exacerbating pain, which in turn often results in cervical ROM limitation, especially limitation of lateral flexion (7). HILT combined with exercise significantly improved lateral flexion but not cervical flexion, extension, rotation. This discrepancy stemmed from anatomical and intervention-specific factors. Lateral flexion primarily involved superficial muscles, which were accessible to laser penetration depth and responsive to laser-induced microcirculation enhancement (24). In contrast, cervical flexion, extension, rotation were highly dependent on the coordinated activation of deep cervical muscles (32), which receive less laser energy due to exponential attenuation with tissue depth (45). Additionally, it may be attributed to factors such as insufficient therapeutic dosage, too short a course of treatment, and only one session of treatment for the subjects recruited in this trial (31).

The findings of this study on improving neck soft tissue stiffness with HILT combined with exercise were consistent with previous similar studies. Szabo et al. (46) found that HILT improved periarticular stiffness in patients with muscular disorders. Ahmad et al. (47) found that HILT combined with exercise appeared to be more effective in improving periarticular soft-tissue stiffness of the knee joints in patients with knee osteoarthritis. HILT group significantly reduced stiffness of superficial cervical muscles (splenius capitis muscle, semispinalis capitis muscle), likely due to its anti-inflammatory and metabolic effects on muscle fibers. The photothermal energy increased collagen elasticity and reduced edema, as evidenced by SWE measurements (48). Conversely, neck fascia stiffness showed no improvement, which may reflect both biological and technical limitations. Fascia, composed of dense connective tissue with fewer mitochondria, was less responsive to photobiomodulation. Moreover, SWE measurements were confined to superficial layers and exhibited directional dependency, failing to capture multi-planar tension patterns in fascia (38, 39). These findings underscore the need for complementary interventions to address fascial dysfunction.

The immediate efficacy of HILT combined with exercise in improving NDI scores in patients with NNP was not significant. The MCID for NDI scores in NNP was established as ≥5 points (28). In this study, the difference between groups in NDI scores did not exceed this threshold. This suggested that functional recovery in NNP required sustained neuromuscular re-education rather than passive modalities alone. The NDI included both physical and psychosocial dimensions, which correlated with deep muscle endurance and cortical motor control—factors unaddressed by a single HILT session (49, 50). Exercise therapy, while effective in acute pain management, must be prolonged to reverse chronic motor control deficits (44). Future protocols should integrate repetitive HILT sessions with progressive exercise dosing to achieve clinically meaningful functional gains.

This study also had some limitations: first, subjects were not included in the study with imaging of the cervical spine to exclude abnormal lesions of the cervical spine; instead, they were first screened by asking whether they had had imaging of the cervical spine in the last 6 months, and then by performing a physical examination if they had had imaging of the cervical spine in the last 6 months and the results were free of any significant abnormality. Then, this clinical trial only investigated the immediate effect of HILT, and all subjects received only one treatment, which was too few sessions, and the results were influenced by subjective factors. Finally, this trial did not measure the stiffness of the deep neck extensor muscles, mainly because the ultrasound diagnostic instrument can only clearly see the superficial neck muscles when switching the shear wave elastography, but cannot clearly show the deep neck muscle groups.

## 5 Conclusion

HILT combined with exercise could immediately improve the pain intensity, cervical lateral flexion mobility, and stiffness of superficial cervical muscles (splenius capitis muscle, semispinalis capitis muscle) in patients with NNP, but has no significant efficacy in immediately improving cervical dysfunction. And more scientific multiple HILT parameters/doses should be set up for studies to find the optimal treatment parameters.

## Data Availability

The raw data supporting the conclusions of this article will be made available by the authors, without undue reservation.

## Footnotes

^1^ http://www.chictr.org.cn/
